# Series Solutions of Three-Dimensional Magnetohydrodynamic Hybrid Nanofluid Flow and Heat Transfer

**DOI:** 10.3390/nano14030316

**Published:** 2024-02-04

**Authors:** Xiangcheng You, Yanbin Wang

**Affiliations:** National Key Laboratory of Petroleum Resources and Engineering, China University of Petroleum (Beijing), Beijing 102249, China; xcyou@cup.edu.cn

**Keywords:** series solutions, three-dimensional, magnetohydrodynamic hybrid nanofluid flow

## Abstract

Hybrid nanofluids have many real-world applications. Research has shown that mixed nanofluids facilitate heat transfer better than nanofluids with one type of nanoparticle. New applications for this type of material include microfluidics, dynamic sealing, and heat dissipation. In this study, we began by placing copper into H_2_O to prepare a Cu-H_2_O nanofluid. Next, Cu-H_2_O was combined with Al_2_O_3_ to create a Cu-Al_2_O_3_-H_2_O hybrid nanofluid. In this article, we present an analytical study of the estimated flows and heat transfer of incompressible three-dimensional magnetohydrodynamic hybrid nanofluids in the boundary layer. The application of similarity transformations converts the interconnected governing partial differential equations of the problem into a set of ordinary differential equations. Utilizing the homotopy analysis method (HAM), a uniformly effective series solution was obtained for the entire spatial region of 0 < *η* < ∞. The errors in the HAM calculation are smaller than 1 × 10^−9^ when compared to the results from the references. The volume fractions of the hybrid nanofluid and magnetic fields have significant impacts on the velocity and temperature profiles. The appearance of magnetic fields can alter the properties of hybrid nanofluids, thereby altering the local reduced friction coefficient and Nusselt numbers. As the volume fractions of nanoparticles increase, the effective viscosity of the hybrid nanofluid typically increases, resulting in an increase in the local skin friction coefficient. The increased interaction between the nanoparticles in the hybrid nanofluid leads to a decrease in the Nusselt number distribution.

## 1. Introduction

With the rapid development of global industrial technologies, the heat transfer load and intensity of heat exchangers are increasing day by day, and the energy consumed during heat transfers is increasing. Shortages of energy have become a major bottleneck, limiting the continued development of industry. Traditional heat exchangers use water, oil, and other cooling media, which have the disadvantage of low efficiency, and the development of macroscopic-scale-enhanced heat transfer technology has reached a certain height and is close to saturation. Therefore, developing new heat transfer refrigerants with high thermal conductivity and good heat transfer performance has become a major focus of current heat transfer technology [1]. The application of thermal energy in the field of petroleum engineering mainly involves the direct utilization or the direct transfer of thermal energy, which is part of the application problem of thermodynamics. Using the principles of heat transfer to select the casing, cement, and various other materials, various oil and gas production and safety processes, among other things, can be determined. For example, the flow and heat transfer laws of nanofluid drilling fluids in the wellbore can be studied to determine the temperature variation in the drilling fluid with the well depth, further determining the composition of the nanofluid drilling fluid.

Alfvén first introduced the term magnetohydrodynamics (MHD) in 1970 [2]. The study of magnetohydrodynamics involves examining the current generated when conductive fluids are in motion and subjected to a magnetic field. This current then applies forces to the ions within conductive fluids. The design of liquid metal refrigeration systems, MHD generators, accelerators, pumps, and flow meters [3,4] can be utilized in various fields. Nanofluids exhibit some new characteristics in porous extended planes; these can significantly improve the heat exchange characteristics of the original base fluids, including microelectronics, fuel cells, pharmaceutical manufacturing, and hybrid locomotives. Nanoparticles serve as a valuable link connecting granular substances and atomic/molecular formations. The involvement of heat exchange is significant in the realms of physics and engineering, ultimately enhancing fluids’ heat exchange properties and bolstering the efficacy of numerous manufacturing procedures [5,6,7,8]. The investigation of the thermal transfer of MHD nanofluids is significant in the fields of physics and engineering. The magnetic field parameters of magnetic fluids (MHD) are one of the key parameters controlling the cooling rate and product quality [9]. Nanofluids have some applications in the polymer and metallurgical industries, such as in handling the stretching of plastic plates and utilizing hydraulic magnetic technology. Nanofluids are utilized to cool microchips, and other electronic applications of microfluidics are seen in the domains of computers and microelectronics [10,11,12]. As superparamagnetic fluids, nanofluids containing magnetic nanoparticles absorb energy and control high temperatures through the interaction of electromagnetic fields. Nanofluids are used as coolants in small-sized and more properly wired heat sinks. Due to their small size, magnetic nanoparticles exhibit behavior similar to compounds in liquids. The fluid contains micron-sized solid particles; magnetic nanomaterials have many advantages not found in traditional heat transfer fluids, such as stable suspension, a high heat transfer coefficient, and resistance to the erosion and blockage of pipelines. When subjected to external magnetic fields, magnetic nanoparticles experience deviation and alignment in the same direction as these magnetic fields, resulting in the formation of various chain-like structures including dimers, trimers, and short chains. The arrangement of these chain-like formations can create a pathway for the heat transfer of magnetic nanofluids, consequently enhancing their thermal conductivity capabilities [13,14,15,16].

Both domestically and internationally, there is a scarcity of research on the utilization of magnetic nanofluids for improved heat transfer. Regarding magnetic nanofluids, especially those under magnetic excitation, experimental, simulation-based, and theoretical research are, both domestically and internationally, still in an immature stage, and there are even differences in the existing research results. The complexity of turbulent convective heat transfer is not well studied, with limited simulation and experimental research conducted on the subject. Using Fe_3_O_4_ nanofluids as the heat transfer medium, Lajvardi et al. [17] studied their heat transfer properties, focusing on convective effects. Furthermore, the concentration of the magnetic nanoparticles and the position of the magnet were analyzed for their effect on heat transfer. The results indicated that increasing the magnetic fields of the fluid concentration could significantly increase the Nusselt number, due to changes in the magnetic field properties; the magnetic fluids changed significantly in terms of their thermal properties. Yarahadi et al. [18] investigated the convective heat transfer of ferromagnetic fluids with concentrations ranging from 1.25% to 2.5%. When the magnetic fields were constant and alternating, a laminar flow (with a Reynolds number ranging from 465 to 1600) was observed. When exposed to magnetic fields, the convective heat transfer coefficient underwent a 12.4% rise.

Raptis and Perdikis [19] investigated the heat transfer of a magnetofluid via laminar convection. They examined the behavior of incompressible viscous conductive fluids under the influence of chemical reactions and magnetic fields. Prasad and Vajravelu [20] conducted a study on the flow of magnetohydrodynamic boundary layers and the heat transfer of power-law fluids on extensional surfaces, specifically focusing on a two-dimensional stable flow on a nonlinear semi-infinite extension plane. Prasad et al. provided numerical solutions [21] for the mixed convective flow of viscous conductive fluids through vertical flat plates in a stable two-dimensional MHD scenario. They assumed that the stretching velocities and transverse magnetic fields were power functions of the distance to the origin. A similar reduction was provided by Hamad et al. [22], who applied a solitary parameter group to address the issue of the magnetic field’s impact on the free convection of semi-infinite flat nanofluids. Using a rotating reference frame, Hamad and Pop [23] investigated the concept of the unsteady magnetohydrodynamic flows of nanofluids on vertical plates that were semi-infinite and experienced permeable oscillation motion, while being subjected to a constant heat source. Hamad [24] obtained analytical solutions for the convection and heat exchange of incompressible viscous nanofluids flowing through a semi-infinite stretching plane under the action of magnetic fields. The Adomian decomposition method (ADM) was employed by Sheikholeslami et al. [25] to examine the impacts of a magnetic field and nanoparticles on Jeffery–Hamel flows. The problem model’s control equations meant that the conventional Navier–Stokes equations and Maxwell electromagnetic equations were simplified into nonlinear ordinary differential equations. Unlike the Runge–Kutta numerical method, this method was able to obtain high-precision results. Using viscous nanofluids, Rosmila et al. [26] studied MHD natural convection and heat transfer flowing through a semi-infinite vertical extension plane, accounting for thermal stratification. The numerical solutions were generated using the Runge–Kutta–Gill method, based on the shooting method. Hamad et al. [27] examined the flow and heat exchange of the boundary layer of a viscous fluid containing metal particles (i.e., a nanofluid) when it flows through a nonlinear stretching plane. This assumes that the rate of stretching is governed by an exponential equation of the distance from the starting point. The problem was numerically solved, obtaining a nonlinear ordinary differential equation.

Farooq et al. [28] analyzed magnetohydrodynamic non-Newtonian Maxwell fluids with nanomaterials that exhibit exponential stretching on the surface. Based on the Buongiorno model, combined with thermal swimming and Brownian motion effects, nonlinear ordinary differential equations were derived from partial differential equations via adequate similarity transformations. BVPh 2.0 was employed to compute local series solutions for extensive control parameters. In the presence of nanoparticles, studies of non-Newtonian Maxwell fluids’ stretching surfaces could be used to obtain the required mass. Xu et al. [29] conducted data analysis using numerical methods on the magnetohydrodynamic (MHD) flows of nanofluids on an extension/contraction wedge. The results indicated that the wedge body only has a unique solution under tension, and, theoretically, there would be no boundary-layer separation. Within a certain range of contraction intensity, there are double solutions, and boundary-layer separation occurs at the wall during the boundary-layer flow. Wall suction could delay boundary-layer separation. The characteristics of the magnetic field had notable effects on the friction coefficient of a stressed wedge, while only slightly influencing the local Nusselt and Sherwood numbers. Numerical simulations were performed by Hao et al. [30], who used the MHD module in Fluent software to conduct convective heat transfer experiments with Fe_3_O_4_ water nanofluids diluted by 3%. The nanofluids were examined under non-uniform magnetic excitations to determine their enhanced heat transfer characteristics. Various vertical uniform magnetic fields were examined with varying magnetic field intensities; the magnetic field intensities remained constant in vertical alternating magnetic fields. The findings of the study indicated that, as the frequency increased, there was a decrease in both the Nusselt number and the convective heat transfer coefficient. Nanofluids with lower Reynolds numbers exhibited a more significant response to changes in the magnetic field frequency. The biological conversion phenomenon of MHD Williamson nanofluids flowing on irregularly thick extension sheets was theoretically studied by Wang et al. [31], who accounted for temperature-dependent non-uniform viscosity and thermal conductivity. A uniformly strong magnetic field produced MHD effects. Ali et al. [32] investigated flows of MHD nanofluids on nonlinear stretchable surfaces of different thicknesses in the presence of electric fields. The findings suggested that the speed of the nanoparticles declined as the strength of the magnetic fields increased. However, as the electric field’s values increased, the temperature of the nanomaterials also increased, as did the velocity distribution. There was an enhancement of the temperature field due to the radiation parameters. When the fluid temperatures rose, the spatial and temporal factors associated with heat generation, absorption, and emission became more evident.

Rajesh et al. [33] investigated energy enhancement using hybrid nanofluids. Their goal was to find an accurate analytical solution for a non-stationary mixed nanofluid with the heat transfer flowing through an infinitely flat vertical plate with a time-varying tilted temperature distribution. To investigate whether thermal radiation and non-uniform heat flux affect the flow of hybrid nanofluids, Ali et al. [34] conducted experiments with magnetohydrodynamics characteristics around a cylinder that was being stretched. According to Jaafar et al. [35], the hybrid nanofluids exhibited steady flows and heat transfer characteristics with nonlinear contracting behavior, in a study that considered the effects of magnetohydrodynamics, thermal radiation, and suction. MHD hybrid nanofluids were examined by Khashi’ie et al. [36], who focused on the movement of a plate with Joule heating. In order to accomplish the study’s objectives, water was utilized as the primary liquid medium, in conjunction with nanoparticles made of metal and metal oxide. Rafique et al. [37] studied the flows of three-dimensional mixed nanofluids on stretched sheets with varying viscosities. Furthermore, the impacts of the Smoluchowski temperature and the implementation of Maxwell velocity slip boundary conditions were also taken into account. Prakash et al. [38] studied the magnetohydrodynamic stagnation flows in the direction of the exponential contraction of thin plates. This research was further enhanced by the inclusion of heat dissipation and thermal radiation. Khashi’ie et al. [36] focused on the movement of a plate undergoing Joule heating, along with the heat transfer of MHD hybrid nanofluids. Their analysis involved the utilization of a mixture comprising copper (Cu) and aluminum oxide (Al_2_O_3_) nanoparticles, with water (H_2_O) used as the underlying liquid. By employing similarity transformation, the complexity of the partial differential equations was diminished to systems of ordinary differential equations. Subsequently, functions of bvp4c in MATLAB were utilized to solve various control parameter values via numerical methods. Lone et al. [39] explored the mixed convection of MHD microelectrode mixed nanofluids through fat surfaces. The flows of a hybrid nanofluid consisting of nanoparticles of alumina and silver were made with water as the base fluid. Suction and injection effects were both experienced by the board when it was positioned vertically in the permeable medium. As well as taking into account viscous dissipation, thermal radiation, and Joule heating, the analysis also considered other factors. Model equations were converted into a dimensionless form using specific similarity variables, and they were solved using the homotopy analysis method (HAM). Roy [40] investigated the convective heat transfer of hybrid nanofluids in an outer shell containing multiple heat sources on the bottom wall. In the study, a magnetic field was applied at a specific angle relative to the horizontal axis in order to observe the natural convection phenomenon. Dimensionless variables and parameters were utilized to systematically establish a set of equations, while also defining flow functions based on velocity components. Subsequently, the solution derived from the finite difference technique was verified using both experimental and numerical data, demonstrating a high level of agreement. Using the proposed model, Alghamdi et al. [41] investigated the influence of MHD on the convection patterns of a heat source and a radiator. The nanofluid was able to consistently exit, purify, compress, and enlarge because the edges of the channels were permeable. Suitable modifications were employed to convert and control partial differential equations and boundary conditions that were relevant to the computations. The researchers utilized the sophisticated HAM to obtain analytical approximations for nonlinear differential equation systems. The main area of study was the smooth movement of mixed copper and copper oxide nanoliquids within a rectangular region between two permeable channels, with blood acting as the fluid that carried them. This method could be used to study drug delivery, flow dynamics, and microcirculation mechanisms. A study carried out by Ramzan et al. [42] examined the movement of carbon-nanotube-based hybrid nanofluids and dust particles suspended in oil on slender needles using the Xue model. In addition, this analysis investigated the impacts of varying thicknesses and Hall currents. The temperature equation was modified by incorporating the Cattaneo–Christov theory and considering the impact of thermal slip-on heat generation for the purpose of conducting a heat transfer analysis. Using the Tiwari Das nanofluid model, a hypothetical mathematical equation was developed. A similarity transformation was applied to convert the control equation for flow into ordinary differential equations. It was determined using bvp4c and the Runge–Kutta shooting method. An investigation conducted by Waini et al. [43] found that vertically contracting thin plates will experience magnetohydrodynamic mixed convection due to thermal radiation. In addition, the influence of Cu and Al_2_O_3_ nanoparticles and dust particles was considered. The control equation was simplified into a similar equation using similar variables and then numerically solved. An experiment conducted by Revnic et al. [44] compared the effects of a magnetic field and heat transfer on a hybrid nanofluid (Cu-Al_2_O_3_–water) inside a square cavity. The walls of the cavity varied in temperature, with the vertical wall being cooler than the middle section of the bottom wall. In the remaining sections of the upper and lower walls, insulation was applied. This study used finite element technology to conduct numerical simulations. The Tiwari Das model was used by Khan et al. [45], who examined how nanoparticles’ shape, viscous dissipation, and nonlinear radiation affect particle behavior. Similarity transformation was used to derive the control equation, and numerical calculations of the flow and temperature fields were performed using MATLAB. The asymptotic tendencies of the high shear strain rate ratio were compared with the numerical solution of the flow field. Ramzan et al. [46] investigated the effect of the flow of a magnetohydrodynamic ternary mixed nanofluid on two different geometric shapes (conical and wedge shaped), taking into account the effects of chemical reactions and thermal radiation. Rafique et al. [47] synthesized ternary hybrid nanoparticles by combining Al_2_O_3_, Cu, and TiO_2_, and then they studied their behavior in the presence of symmetric stretching discs in H_2_O. They analyzed the effects of many parameters on coolant applications, including the MHD stagnation flow, ternary mixed nanofluids, viscous dissipation, variable viscosity, thermal stratification, and velocity slip conditions.

## 2. Mathematical Description

**Figure 1:**
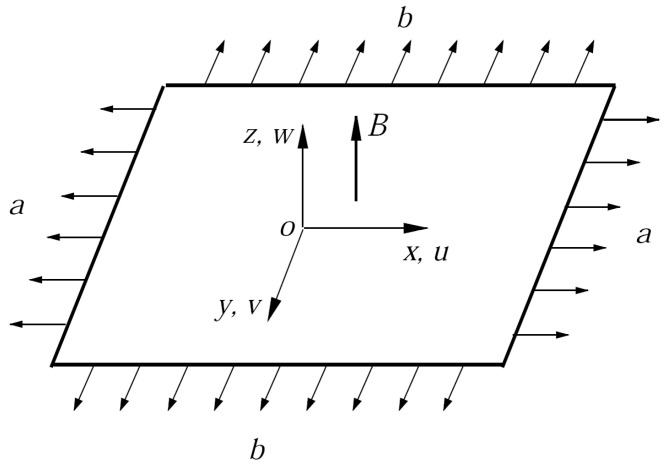
Coordinate system.

Consider steady, three-dimensional mixed convection flows of nanofluids past stretching sheets in the presence of an applied magnetic field. A schematic diagram of a physical model and a coordinate system is shown in Figure 1. Here, consider three different types of nanoparticles: Cu and Al_2_O_3_ are shown in Table 1. Assuming that hybrid nanofluids are incompressible and the flows are laminar, the velocities of the stretching sheets are $u_{w}=ax$ in *x* and $v_{w}=by$ in *y*, and a uniform external magnetic field B is applied in *z*. The surface temperature has a constant value of $T_{w}$ and the ambient temperature is $T_{\infty }$ , where $T_{w}$ > $T_{\infty }$ . Using the hybrid nanofluid model proposed by Wainia et al. [48] and referring to Xu and Zhao et al. [49,50], the governing equations are given as follows:

(1) $$\frac{\partial u}{\partial x}+\frac{\partial v}{\partial y}+\frac{\partial w}{\partial z}=0,$$

(2) $$u\frac{\partial u}{\partial x}+v\frac{\partial u}{\partial y}+w\frac{\partial u}{\partial z}=\frac{\mu_{hnf}}{\rho_{hnf}}\frac{\partial^{2}u}{\partial z^{2}}-\frac{\sigma_{hnf}B^{2}u}{\rho_{hnf}},$$

(3) $$u\frac{\partial v}{\partial x}+v\frac{\partial v}{\partial y}+w\frac{\partial v}{\partial z}=\frac{\mu_{hnf}}{\rho_{hnf}}\frac{\partial^{2}v}{\partial z^{2}}-\frac{\sigma_{hnf}B^{2}v}{\rho_{hnf}},$$

(4) $$u\frac{\partial T}{\partial x}+v\frac{\partial T}{\partial y}+w\frac{\partial T}{\partial z}=\alpha_{hnf}\frac{\partial^{2}T}{\partial z^{2}}.$$

They are subject to the following boundary conditions:

$$u\left(x,y,0\right)=u_{w}=ax,\;v\left(x,y,0\right)=v_{w}=by,\;w\left(x,y,0\right)=0,$$

(5) $$u\left(x,y,\infty \right)=v\left(x,y,\infty \right)=0,\;T\left(x,y,0\right)=T_{w},\;T\left(x,y,\infty \right)=T_{\infty },$$

where $\left(u,v,w\right)$ are the velocity components in axes $\left(x,y,z\right)$ , T is the temperature of the hybrid nanofluid, σ is the electrical conductivity, and a , b , B represent positive constants. $\mu_{hnf}$ , $\rho_{hnf}$ , $\alpha_{hnf}$ are the effective hybrid nanofluid viscosity, and they are defined as follows:

$$\mu_{hnf}=\frac{\mu_{f}}{{\left(1-\phi_{1}\right)}^{2.5}{\left(1-\phi_{2}\right)}^{2.5}},\;\alpha_{hnf}=\frac{k_{hnf}}{{\left(\rho C_{p}\right)}_{hnf}},$$

$$\rho_{hnf}=(1-\phi_{2})[(1-\phi_{1})\rho_{f}+\phi_{1}\rho_{n1}]+\phi_{2}\rho_{n2},$$

$${\left(\rho C_{p}\right)}_{hnf}=(1-\phi_{2})[(1-\phi_{1}){\left(\rho C_{p}\right)}_{f}+\phi_{1}{\left(\rho C_{p}\right)}_{n1}]+\phi_{2}{\left(\rho C_{p}\right)}_{n2},$$

$$\frac{k_{hnf}}{k_{nf}}=\frac{k_{n2}+2k_{nf}-2\phi_{2}\left(k_{nf}-k_{n2}\right)}{k_{n2}+2k_{nf}+\phi_{2}\left(k_{nf}-k_{n2}\right)},\;\frac{k_{nf}}{k_{f}}=\frac{k_{n1}+2k_{f}-2\phi_{1}\left(k_{f}-k_{n1}\right)}{k_{n1}+2k_{f}+\phi_{1}\left(k_{f}-k_{n1}\right)},$$

(6) $$\frac{\sigma_{hnf}}{\sigma_{nf}}=\frac{\sigma_{n2}+2\sigma_{nf}-2\phi_{2}\left(\sigma_{nf}-\sigma_{n2}\right)}{\sigma_{n2}+2\sigma_{nf}+\phi_{2}\left(\sigma_{nf}-\sigma_{n2}\right)},\;\frac{\sigma_{nf}}{\sigma_{f}}=\frac{\sigma_{n1}+2\sigma_{f}-2\phi_{1}\left(\sigma_{f}-\sigma_{n1}\right)}{\sigma_{n1}+2\sigma_{f}+\phi_{1}\left(\sigma_{f}-\sigma_{n1}\right)}.$$

$$\mu_{tnf}=\frac{\mu_{f}}{{\left(1-\phi_{1}\right)}^{2.5}{\left(1-\phi_{2}\right)}^{2.5}{\left(1-\phi_{3}\right)}^{2.5}},\;\alpha_{tnf}=\frac{k_{tnf}}{{\left(\rho C_{p}\right)}_{tnf}},$$

$$\rho_{tnf}=(1-\phi_{3})\left\{(1-\phi_{2})[(1-\phi_{1})\rho_{f}+\phi_{1}\rho_{n1}]+\phi_{2}\rho_{n2}\right\}+\phi_{3}\rho_{n3},$$

$${\left(\rho C_{p}\right)}_{tnf}=(1-\phi_{3})\left\{(1-\phi_{2})[(1-\phi_{1}){\left(\rho C_{p}\right)}_{f}+\phi_{1}{\left(\rho C_{p}\right)}_{n1}]+\phi_{2}{\left(\rho C_{p}\right)}_{n2}\right\}+\phi_{3}{\left(\rho C_{p}\right)}_{n3},$$

$$\frac{k_{tnf}}{k_{hnf}}=\frac{k_{n3}+2k_{hnf}-2\phi_{3}\left(k_{hnf}-k_{n3}\right)}{k_{n3}+2k_{hnf}+\phi_{3}\left(k_{hnf}-k_{n3}\right)},$$

$$\frac{k_{hnf}}{k_{nf}}=\frac{k_{n2}+2k_{nf}-2\phi_{2}\left(k_{nf}-k_{n2}\right)}{k_{n2}+2k_{nf}+\phi_{2}\left(k_{nf}-k_{n2}\right)},\;\frac{k_{nf}}{k_{f}}=\frac{k_{n1}+2k_{f}-2\phi_{1}\left(k_{f}-k_{n1}\right)}{k_{n1}+2k_{f}+\phi_{1}\left(k_{f}-k_{n1}\right)},$$

$$\frac{\sigma_{tnf}}{\sigma_{hnf}}=\frac{\sigma_{n3}+2\sigma_{hnf}-2\phi_{3}\left(\sigma_{hnf}-\sigma_{n3}\right)}{\sigma_{n3}+2\sigma_{hnf}+\phi_{3}\left(\sigma_{hnf}-\sigma_{n3}\right)},$$

(7) $$\frac{\sigma_{hnf}}{\sigma_{nf}}=\frac{\sigma_{n2}+2\sigma_{nf}-2\phi_{2}\left(\sigma_{nf}-\sigma_{n2}\right)}{\sigma_{n2}+2\sigma_{nf}+\phi_{2}\left(\sigma_{nf}-\sigma_{n2}\right)},\;\frac{\sigma_{nf}}{\sigma_{f}}=\frac{\sigma_{n1}+2\sigma_{f}-2\phi_{1}\left(\sigma_{f}-\sigma_{n1}\right)}{\sigma_{n1}+2\sigma_{f}+\phi_{1}\left(\sigma_{f}-\sigma_{n1}\right)}.$$

where $\mu_{f}$ is the base fluid viscosity, $\phi_{1}$ , $\phi_{2}$ , $\phi_{3}$ are the hybrid nanofluid volume fractions of Cu-Al_2_O_3_-TiO_2_-H_2_O, ρ is the density, k is the thermal conductivity, $\rho C_{p}$ is the heat capacitance, $\sigma_{hnf}$ is the electrical conductivity of hybrid nanofluid [40], $\sigma_{tnf}$ is the electrical conductivity of the ternary hybrid nanofluid [46,47], the subscript tnf means ternary hybrid nanofluid, hnf means hybrid nanofluid, nf means nanofluid, f means base fluid, and n means nanoparticle. $\mu_{nf}$ in Equation (6) is obtained from [52], Equation (7) is obtained from [46,47], and $k_{nf}$ is assumed by the Maxwell–Garnett model [53]. Additionally, only spherical nanoparticles are considered in terms of their shape.

The subsequent similarity conversions are as follows:

$$\eta =z\sqrt{\frac{a}{\nu_{f}}},\;u\left(x,y,z\right)=axf^{\prime }\left(\eta \right),\;v\left(x,y,z\right)=ayg^{\prime }\left(\eta \right),$$

(8) $$w\left(x,y,z\right)=-\sqrt{a\nu_{f}}\left(f+g\right),\;T\left(x,y,z\right)=T_{\infty }+\left(T_{w}-T_{\infty }\right)s\left(\eta \right).$$

Equations (2)–(4) are simplified accordingly:

(9) $$\zeta_{1}f^{\prime \prime \prime }\left(\eta \right)+f^{\prime \prime }\left(\eta \right)\left[f\left(\eta \right)+g\left(\eta \right)\right]-{\left[f^{\prime }\left(\eta \right)\right]}^{2}-Mf^{\prime }\left(\eta \right)=0,$$

(10) $$\zeta_{1}g^{\prime \prime \prime }\left(\eta \right)+g^{\prime \prime }\left(\eta \right)\left[f\left(\eta \right)+g\left(\eta \right)\right]-{\left[g^{\prime }\left(\eta \right)\right]}^{2}-Mg^{\prime }\left(\eta \right)=0,$$

(11) $$\frac{\zeta_{2}}{\mathrm{Pr}}s^{\prime \prime }\left(\eta \right)+s^{\prime }\left(\eta \right)\left[f\left(\eta \right)+g\left(\eta \right)\right]=0.$$

The dimensionless boundary conditions are as follows:

(12) $$f\left(0\right)=0,\;f^{\prime }\left(0\right)=1,\;f^{\prime }\left(\infty \right)=0,$$

(13) $$g\left(0\right)=0,\;g^{\prime }\left(0\right)=c,\;g^{\prime }\left(\infty \right)=0,$$

(14) $$s\left(0\right)=1,\;s\left(\infty \right)=0,$$

where

(15) $$M=\frac{\sigma_{hnf}B^{2}}{\rho_{hnf}a},\;\mathrm{Pr}=\frac{\nu_{f}}{\alpha_{hnf}},\;c=\frac{b}{a}\geq 0,$$

(16) $$\zeta_{1}=\frac{1}{{\left(1-\phi_{1}\right)}^{2.5}{\left(1-\phi_{2}\right)}^{2.5}\rho_{hnf}/\rho_{f}},\;\zeta_{2}=\frac{k_{hnf}/k_{f}}{{\left(\rho C_{p}\right)}_{hnf}/{\left(\rho C_{p}\right)}_{f}}.$$

Skin friction coefficients along *x* and *y* and the Nusselt number can be expressed as follows:

(17) $$C_{fx}=\frac{\tau_{x}}{\rho_{f}u_{w}^{2}},\;C_{fy}=\frac{\tau_{y}}{\rho_{f}u_{w}^{2}},\;Nu=\frac{xq_{w}}{k_{f}\left(T_{w}-T_{\infty }\right)},$$

where $\tau_{x}$ , $\tau_{y}$ refer to the shear stresses in the *x*, *y* directions, and q is the heat flux from the stretching sheets, which is given by

(18) $$\tau_{x}=-\mu_{hnf}{\left(\frac{\partial u}{\partial z}\right)}_{z=0},\;\tau_{y}=-\mu_{hnf}{\left(\frac{\partial v}{\partial z}\right)}_{z=0},\;q_{w}=-k_{hnf}{\left(\frac{\partial T}{\partial z}\right)}_{z=0}.$$

Using (8), (17) and (18), we obtain the following:

Cfx=−Rex−1/21−ϕ12.51−ϕ22.5f″0, Cfy=−Rey−1/2c1.51−ϕ12.51−ϕ22.5g″0,

(19) Nux=−Rex1/2khnfkfs′0, Nuy=−Rey1/2khnfc0.5kfs′0,

in which Rex=uwx/νf and Rey=vwy/νf are local Reynolds numbers.

## 3. Asymptotic Analysis and Results

Following Takhar [54], we investigate the asymptotic behavior of $f^{\prime }$ , $g^{\prime }$ , and s at infinity. For large η , $f^{\prime }\to 0$ , g′→0 , s→0 , and the boundary conditions of (12)–(14), we obtain

(20) $$\underset{\eta \to \infty }{\lim}f\to \alpha_{1},\;\underset{\eta \to \infty }{\lim}g\to \alpha_{2}.$$

For large η , suppose that F , G , S are small, and f , g , s are expressed as

(21) $$f=\alpha_{1}+F,\;g=\alpha_{2}+G,\;s=S,$$

and

(22) $$\alpha_{3}=\alpha_{1}+\alpha_{2}.$$

By linearizing Equations (9)–(11), we obtain

(23) $$\zeta_{1}F\prime \prime \prime +\alpha_{3}F\prime \prime \prime MF^{\prime }=0,$$

(24) $$\zeta_{1}G\prime \prime \prime +\alpha_{3}G^{\prime \prime \prime }MG^{\prime }=0,$$

(25) $$\frac{\zeta_{2}}{\mathrm{Pr}}S^{\prime \prime \prime }+\alpha_{3}S^{\prime }=0.$$

Due to (21), the boundary conditions are given by

(26) $$F^{\prime }=G^{\prime }=S=0\;as\;\eta \to \infty .$$

Combined with the boundary conditions (26), we obtain

(27) $$F^{\prime }=G^{\prime }=B_{1}\exp\left(-\lambda_{1}\eta \right),\;S=B_{2}\exp\left(-\lambda_{2}\eta \right),$$

where $\lambda_{1}=\frac{\alpha_{3}+\sqrt{\alpha_{3}^{2}+4\varepsilon_{1}M}}{2\zeta_{1}}$ , $\lambda_{2}=\frac{\alpha_{3}\mathrm{Pr}}{\zeta_{2}}$ , and $B_{1}$ and $B_{2}$ are some arbitrary constants. If $\alpha_{3}>0$ , $F^{\prime }$ , $G^{\prime }$ , S (or $f^{\prime }$ , $g^{\prime }$ , s ), there is exponential decay to zero as η→∞ .

Using the homotopy analysis method (HAM) [55], Equations (9)–(11) can be solved with the boundary conditions (12)–(14). There is extensive literature introducing the analytical technique and its applications; hence, we provide only the necessary information regarding the HAM process. From a physics perspective, $f^{\prime }$ , $g^{\prime }$ , and s represent the reduced velocity and temperature; therefore, $\alpha_{3}>0$ always holds. It is known that most of the boundary-layer problems decay exponentially. Therefore, the solution should contain the term $\exp\left(-n\eta \right)$ , n≥1 . $f\left(\eta \right)$ , $g\left(\eta \right)$ , and $s\left(\eta \right)$ can be expressed as follows:

(28) $$\left\{\eta^{m}\left.e^{-n\lambda \eta }\right|m\geq 0,n\geq 0\right\},$$

(29) $$f\left(\eta \right)=\sum_{m=0}^{+\infty }\sum_{n=0}^{+\infty }a_{m,n}\eta^{m}e^{-n\lambda \eta },\;g\left(\eta \right)=\sum_{m=0}^{+\infty }\sum_{n=0}^{+\infty }b_{m,n}\eta^{m}e^{-n\lambda \eta },$$

(30) $$s\left(\eta \right)=\sum_{m=0}^{+\infty }\sum_{n=0}^{+\infty }c_{m,n}\eta^{m}e^{-n\lambda \eta },$$

where $a_{m,n}$ , $b_{m,n}$ , and $c_{m,n}$ are coefficients, and λ is a spatial-scale parameter. Based on the rules of the solution expressions and the boundary conditions (12)–(14), we select the following as initial approximations:

(31) $$f_{0}\left(\eta \right)=\frac{1-e^{-\lambda \eta }}{\lambda },\;g_{0}\left(\eta \right)=\frac{c\left(1-e^{-\lambda \eta }\right)}{\lambda },$$

(32) $$s_{0}\left(\eta \right)=e^{-\lambda \eta }.$$

Auxiliary linear operators are chosen in the following manner:

(33) $$L_{f}\left[\phi \right]=\frac{\partial^{3}\phi }{\partial \eta^{3}}-\lambda^{2}\frac{\partial \phi }{\partial \eta },\;L_{g}\left[\gamma \right]=\frac{\partial^{3}\gamma }{\partial \eta^{3}}-\lambda^{2}\frac{\partial \gamma }{\partial \eta },$$

(34) $$L_{s}\left[\theta \right]=\frac{\partial^{2}\theta }{\partial \eta^{2}}-\lambda^{2}\theta ,$$

with the definition of three residual error functions as follows:

(35) $$Err_{1}={\int_{0}^{\infty }\left[\zeta_{1}f^{\prime \prime \prime }+\left(f+g\right)f^{\prime \prime }-{\left(f^{\prime }\right)}^{2}-Mf\prime \right]}^{2}d\eta ,$$

(36) $$Err_{2}={\int_{0}^{\infty }\left[\zeta_{1}g^{\prime \prime \prime }+\left(f+g\right)g\prime \prime -{\left(g^{\prime }\right)}^{2}-Mg^{\prime }\right]}^{2}d\eta ,$$

(37) $$Err_{3}={\int_{0}^{\infty }\left[\frac{\zeta_{2}}{\mathrm{Pr}}s^{\prime \prime \prime }+\left(f+g\right)s^{\prime }\right]}^{2}d\eta .$$

The advantages of HAM technology over other methods are as follows [46,56,57]: HAM methods are used for weak and strong nonlinear problems. Moreover, HAM technology is independent of size constraints. Using HAM, any nonlinear partial differential equation system can be solved without linearization and discretization. By using HAM technology, the convergence solution and series solution of the system were obtained. In order to obtain a series solution, the homotopy analysis method was used; it is uniformly effective across the entire spatial region 0 < *η* < ∞. When $\phi_{1}=\phi_{2}=0$ of the hybrid nanofluid Cu-Al_2_O_3_-H_2_O, M=0 , Pr=1 , the comparison of the 20th-order HAM determined the parameters λ=1 , ℏ=−0.7 with reference to Wang’s data [58]; the results are shown in Table 2. In this special case of the Newtonian fluid of water, we observe the three-dimensional fluid motion caused by the stretching of the plane boundary. The series solution obtained using the homotopy analysis method compares favorably with the results from the literature. The HAM residual errors are less than 1 × 10^−7^. As the volume fractions of the nanoparticles $\phi_{1}$ , $\phi_{2}$ , $\phi_{3}$ increase, the series solutions of the velocity profiles and temperature distributions are impacted. The shapes of nanoparticles also affect the calculation results, and only spherical particles are considered in this study.

**Figure 2:**
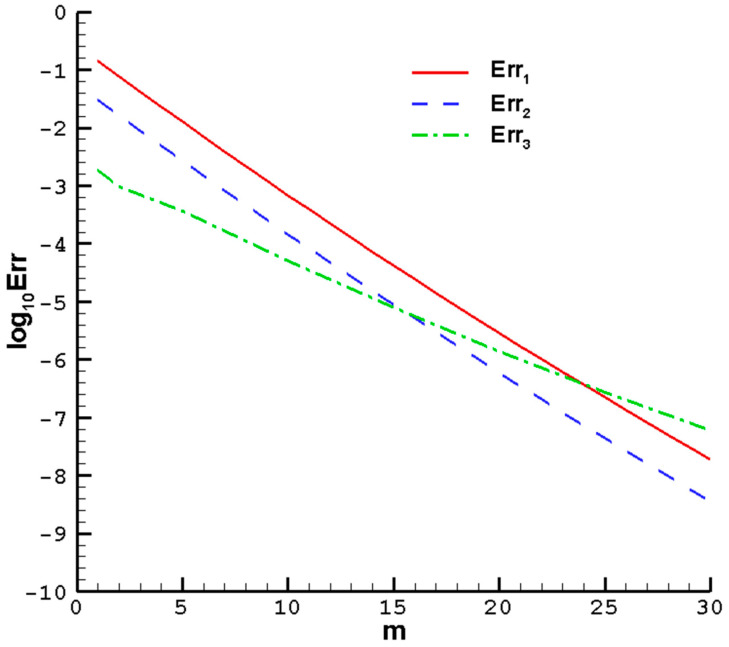
The HAM residual errors of $\phi_{1}=0.1$ , $\phi_{2}=0$ of the hybrid nanofluid Cu-Al_2_O_3_-H_2_O; M=1 , Pr=1 , c=0.5 , determined parameters λ=0.6 , ℏ=−0.35 . *Err*_1_—solid line; *Err*_2_—dashed line; *Err*_3_—dashed dotted line.

**Figure 3:**
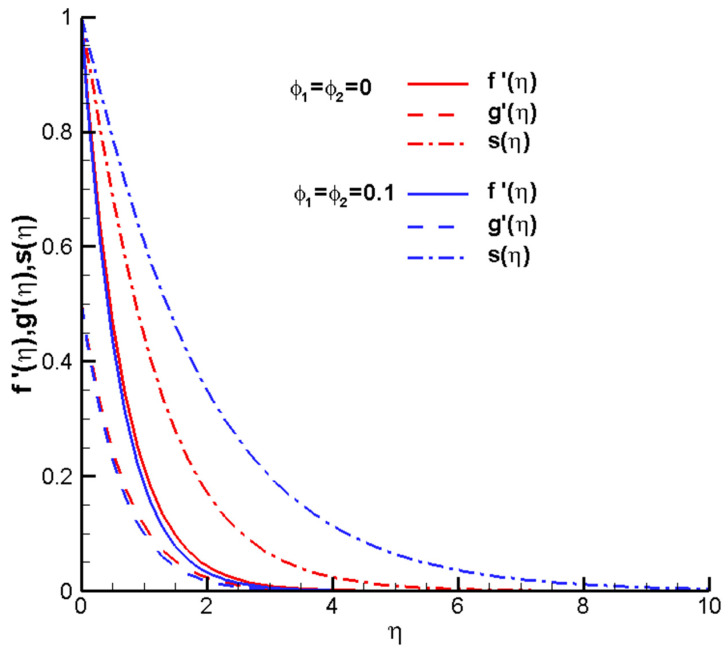
Twentieth-order HAM results of the hybrid nanofluid Cu-Al_2_O_3_-H_2_O when $\phi_{1}=\phi_{2}=$ 0, 0.1, M=1 , Pr=1 , c=0.5 , determined parameters λ=1 , ℏ=−0.8 . $f^{\prime }\left(\eta \right)$ —solid line; $g\prime \left(\eta \right)$ —dashed line; $s\left(\eta \right)$ —dashed dots line.

**Figure 4:**
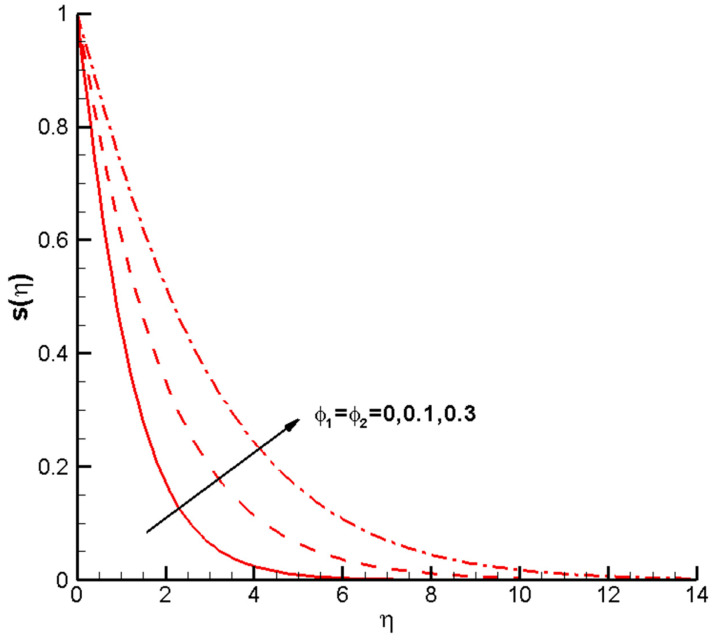
Twentieth-order HAM temperature profiles $s\left(\eta \right)$ of the hybrid nanofluid Cu-Al_2_O_3_-H_2_O when $\phi_{1}=\phi_{2}=$ 0, 0.1, 0.3, M=1 , Pr=1 , c=0.5 , determined parameters λ=1 , ℏ=−0.8 .

As shown in Figure 2, when $\phi_{1}=0.1$ , $\phi_{2}=0$ of the hybrid nanofluid Cu-Al_2_O_3_-H_2_O, with Pr=1 , c=0.5 , and the determined parameters λ=0.6 , ℏ=−0.35 , the series solution obtained is uniformly effective in the various orders of HAM computation. Residual errors for the mth-order HAM computation and CPU times (Lenovo P720) are shown in Table 3. For the first-order HAM, the residual errors are $Err_{1}$ = 0.15849, $Err_{2}$ = 0.15849, and $Err_{3}$ = 0.00251. As the mth order of the HAM-approximated analytical solution increases, the residual errors gradually decrease. When *m* < 15, the relationship of the three residual errors is $Err_{1}$ > $Err_{2}$ > $Err_{3}$ . For the fifteenth-order HAM, the residual errors are between 3.98107 × 10^−5^ and 7.94328 × 10^−6^, with CPU times of 127.641 s. Then, $Err_{3}$ begins to exceed $Err_{2}$ . When *m* > 25, the relationship of the three residual errors is $Err_{3}$ > $Err_{1}$ > $Err_{2}$ . Compared with the hybrid nanofluid flow equations, the convergence speed of the coupled temperature equation is slower. For the 30th-order HAM, the residual errors are between 1.62181 × 10^−8^ and 3.98107 × 10^−9^, with CPU times of 4006.94 s (more than 30 times the 15th-order calculation). $\phi_{1}$ , $\phi_{2}$ describe the physical quantity of the volume fraction of the solid Cu and Al_2_O_3_ nanoparticles in the hybrid nanofluid Cu-Al_2_O_3_-H_2_O. $\phi_{1}=\phi_{2}=0$ represents the Newtonian fluid of water, and $\phi_{1}$ , $\phi_{2}$ ≥ 0 represents hybrid nanofluids. An analysis of the influence of the nanoparticle volume fraction $\phi_{1}$ , $\phi_{2}$ on the velocity profiles of $f^{\prime }\left(\eta \right)$ , $g^{\prime }\left(\eta \right)$ and temperature distributions $s\left(\eta \right)$ is shown in Figure 3. The solid lines represent velocity profiles $f\prime \left(\eta \right)$ , the dashed lines show the velocity profiles $g\prime \left(\eta \right)$ , and the dashed dots show the temperature profiles $s\left(\eta \right)$ . When $\phi_{1}$ , $\phi_{2}$ increases from 0 to 0.1, the velocity profiles $f^{\prime }\left(\eta \right)$ , $g\prime \left(\eta \right)$ decrease. As the concentration of nanoparticles increases, a decrease in velocity can be observed. As shown in Figure 4, when $\phi_{1}$ , $\phi_{2}$ increases from 0 to 0.1, the temperature profile $s\left(\eta \right)$ increases. Changes in velocity follow a similar trend; the increase in $s\left(\eta \right)$ monotonically decreases along with η . The larger the volume fraction of the nanoparticles $\phi_{1}$ , $\phi_{2}$ , the higher the temperature distribution $s\left(\eta \right)$ . This can be attributed to the hybrid nanofluids, which possess better thermal conductivity than the base fluid (water). Consequently, the fluid’s heat transfer capacity is significantly enhanced, resulting in an improved distribution of temperature. The findings suggest that, with an increase in the proportion of nanoparticles in the hybrid nanofluid Cu-Al_2_O_3_-H_2_O, there is a decrease in the velocity distribution, an increase in the temperature distribution, and the boundary layer’s thickness becomes noticeably more prominent. The calculation results indicate that, as the volume percentage increases, the velocity profile shows a downward trend, and the temperature curve shows an upward trend. A larger volume fraction is responsible for the increased viscosity of the nanofluids.

**Figure 5:**
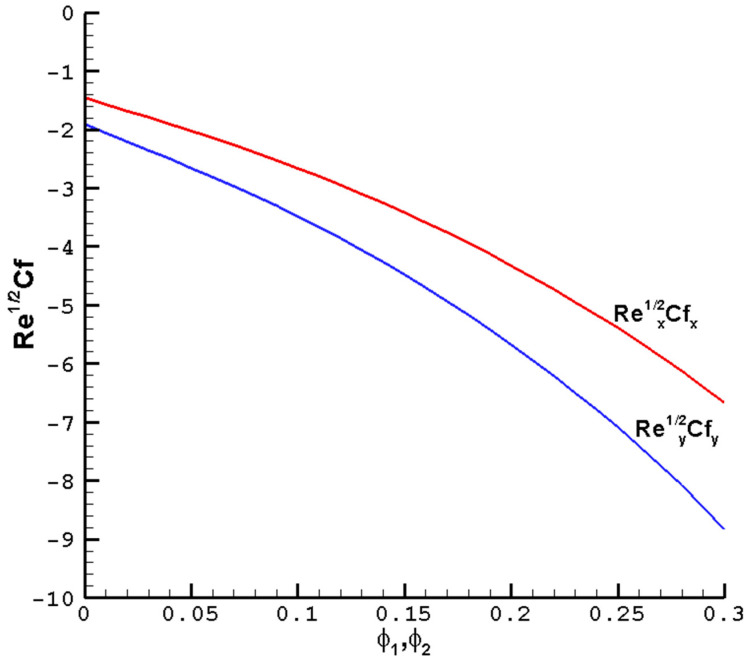
The local reduced friction coefficient Rex1/2Cfx , Rey1/2Cfy varies with the $\phi_{1}$ , $\phi_{2}$ of the hybrid nanofluid Cu-Al_2_O_3_-H_2_O when M=1 , Pr=1 , c=0.5 , and the determined parameters λ=1 , ℏ=−0.8 .

**Figure 6:**
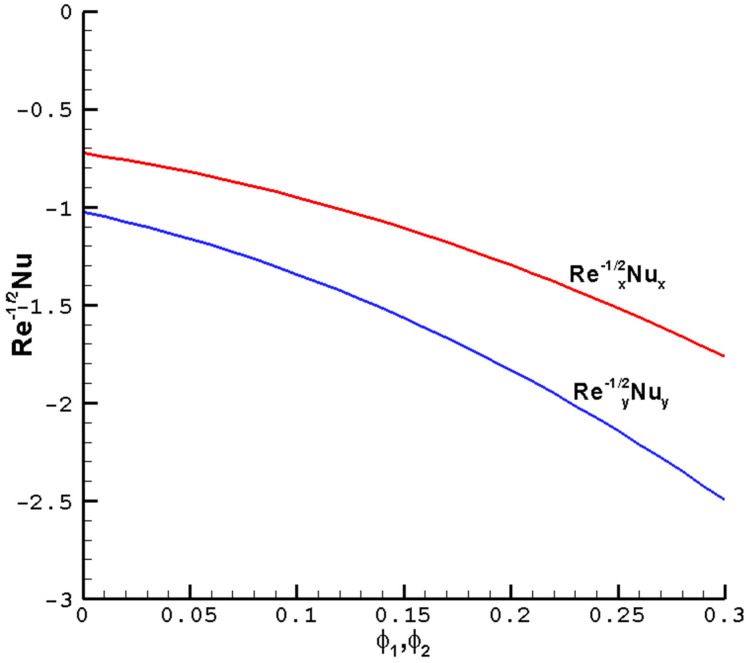
The local reduced Nusselt number Rex−1/2Nux , Rey−1/2Nuy vary with the $\phi_{1}$ , $\phi_{2}$ of the hybrid nanofluid Cu-Al_2_O_3_-H_2_O when M=1 , Pr=1 , c=0.5 , and the determined parameters λ=1 , ℏ=−0.8 .

Figure 5 considers the effects of the nanoparticle volume fraction on the $\phi_{1}$ , $\phi_{2}$ of the local skin friction coefficient and the local Nusselt number. As the $\phi_{1}$ , $\phi_{2}$ of the hybrid nanofluid Cu-Al_2_O_3_-H_2_O shifts from 0 to 0.3, the reduced local skin friction coefficients Rex1/2Cfx and Rey1/2Cfy both decrease monotonically. As the volume fractions of the nanoparticles $\phi_{1}$ , $\phi_{2}$ increase, the local skin friction coefficients decrease, while the heat transfer increases. Notably, the nanoparticles exhibit a greater amount of kinetic energy when their concentrations are higher, resulting in an enhancement of their heat transfer ability through increased kinetic energy. Similarly to the variation trend of the local skin friction coefficients, the local Nusselt numbers Rex−1/2Nux and Rey−1/2Nuy vary with $\phi_{1}$ , $\phi_{2}$ . Figure 6 shows that the local Nusselt numbers experience a gradual decrease and evolve at a slower pace than the local skin friction coefficients. This can be attributed to the volume fraction of the nanoparticles $\phi_{1}$ , $\phi_{2}$ of the hybrid nanofluid Cu-Al_2_O_3_-H_2_O. The convective and conductive heat transfer ratios across the boundary can be increased by enhancing the heat transfer. Given the superior thermal conductivity and heat transfer abilities of nanofluids over pure fluids, this phenomenon once again proves that nanofluids are superior to pure fluids.

**Figure 7:**
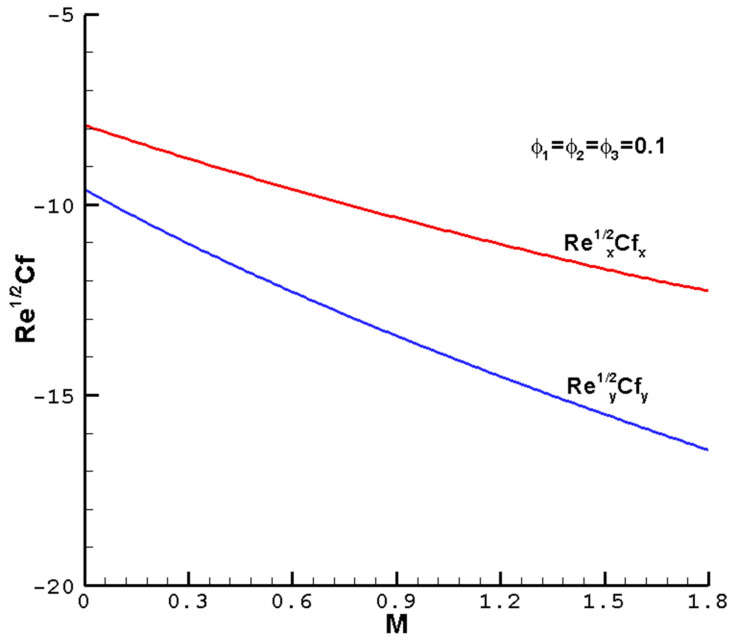
The local reduced friction coefficient Rex1/2Cfx , Rey1/2Cfy varies with the M of ternary hybrid nanofluid Cu-Al_2_O_3_-TiO_2_-H_2_O when $\phi_{1}=\phi_{2}=\phi_{3}$ = 0.1, Pr=1 , c=0.5 , and the determined parameters λ=1 , ℏ=−0.8 .

**Figure 8:**
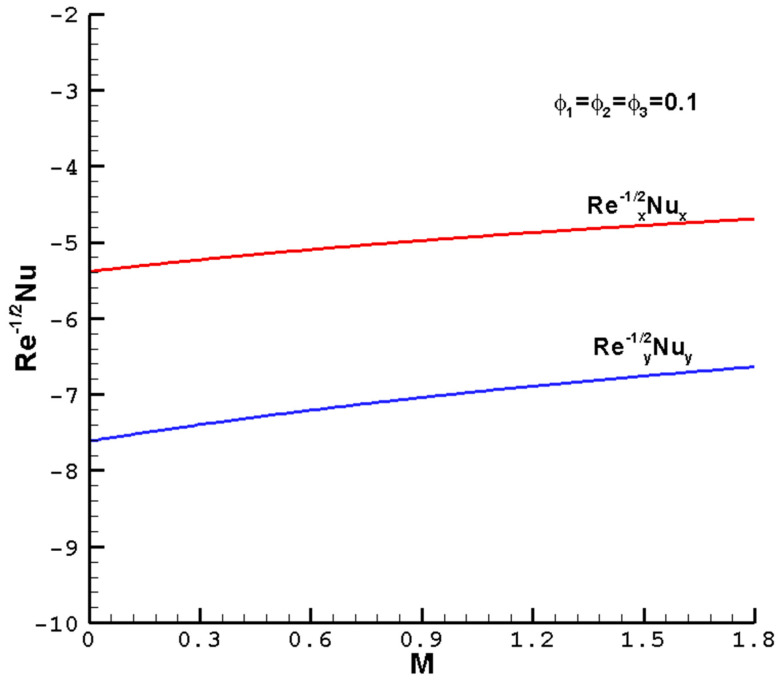
The local reduced Nusselt numbers Rex−1/2Nux , Rey−1/2Nuy vary with the M of the ternary hybrid nanofluid Cu-Al_2_O_3_-TiO_2_-H_2_O when $\phi_{1}=\phi_{2}=\phi_{3}$ = 0.1, Pr=1 , c=0.5 , and the determined parameters λ=1 , ℏ=−0.8 .

**Figure 9:**
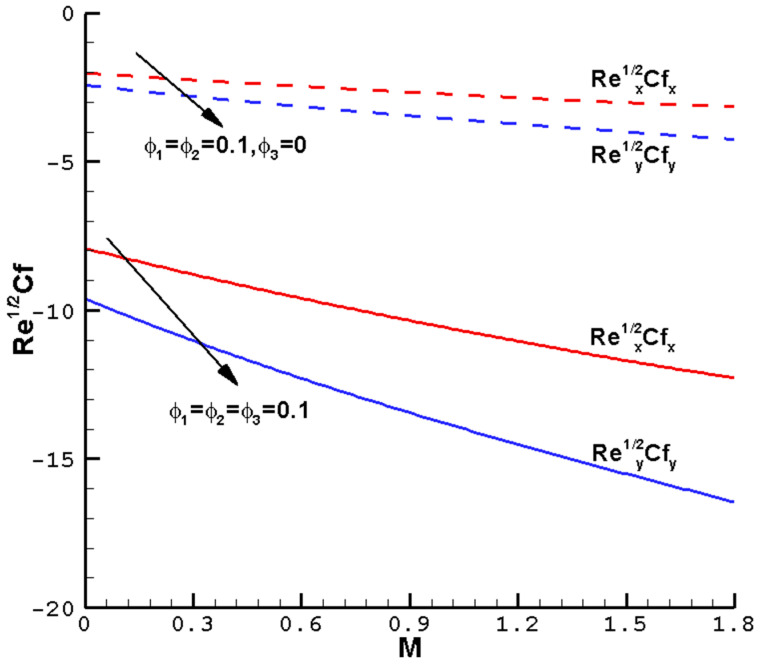
The local reduced friction coefficient Rex1/2Cfx , Rey1/2Cfy varies with the M of the ternary hybrid nanofluid Cu-Al_2_O_3_-TiO_2_-H_2_O when Pr=1 , c=0.5 , and the determined parameters are λ=1 , ℏ=−0.8 .

**Figure 10:**
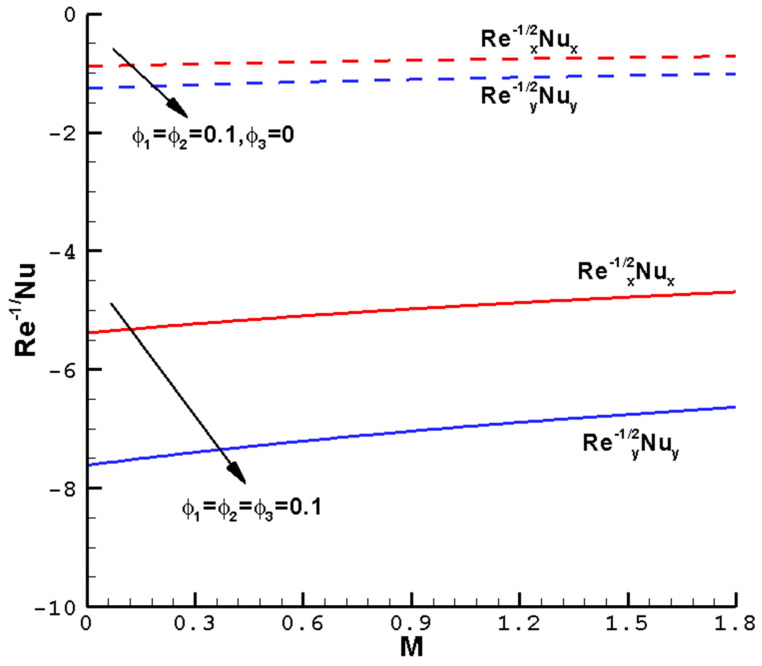
The local reduced Nusselt numbers Rex−1/2Nux , Rey−1/2Nuy vary with the M of the ternary hybrid nanofluid Cu-Al_2_O_3_-TiO_2_-H_2_O when Pr=1 , c=0.5 , and the determined parameters are λ=1 , ℏ=−0.8 .

Figure 7, Figure 8, Figure 9 and Figure 10 show the effects of the different parameters $\phi_{1}$ , $\phi_{2}$ , $\phi_{3}$ , M on physical measurements, such as the skin damage and heat transfer rates at specific locations. The most important industrial parameters are the local reduced friction coefficients Rex1/2Cfx , Rey1/2Cfy and the Nusselt numbers Rex−1/2Nux and Rey−1/2Nuy . When a fluid flows on a surface, the distribution of the frictional force applied to the surface is called the skin friction profile of the fluid. The ratio of these frictional forces to the fluid dynamic pressure should reflect the skin friction coefficient. By applying a magnetic field to a fluid, magnetohydrodynamic (MHD) effects can be induced in the fluid. The appearance of magnetic fields can alter the properties of hybrid nanofluids, thereby altering the local reduced friction coefficient and the Nusselt numbers. Figure 7 illustrates the effect of the magnetic parameter M of the ternary hybrid nanofluid Cu-Al_2_O_3_-TiO_2_-H_2_O on Rex1/2Cfx and Rey1/2Cfy when $\phi_{1}=\phi_{2}=\phi_{3}$ = 0.1, Pr=1 , and c=0.5 , and the determined parameters λ=1 and ℏ=−0.8 . As M increases from 0 to 1.8, Rex1/2Cfx increases by 54.89%, and Rey1/2Cfy increases by 71.10%. As the magnetic parameters increase, the arranged nanoparticles interact with the boundary layer in a more robust manner. Due to this contact, the shear stress may increase; therefore, the skin friction profile of the hybrid nanofluids increases with the increase in the magnetic parameter values. Figure 8 shows the effect of the magnetic parameter M of the ternary hybrid nanofluid Cu-Al_2_O_3_-TiO_2_-H_2_O on Rex−1/2Nux and Rey−1/2Nuy when $\phi_{1}=\phi_{2}=\phi_{3}$ = 0.1, Pr=1 , c=0.5 , and the determined parameters λ=1 and ℏ=−0.8 . As M increases from 0 to 1.8, Rex−1/2Nux and Rey−1/2Nuy decrease by 12.87%. Figure 9 illustrates the effects of the magnetic parameter M and the volume fractions $\phi_{1},\phi_{2},\phi_{3}$ on Rex1/2Cfx and Rey1/2Cfy when Pr=1 , c=0.5 , and the determined parameters λ=1 and ℏ=−0.8 . As $\phi_{3}$ increases from 0 to 0.1, Rex1/2Cfx and Rey1/2Cfy increase significantly. Figure 10 shows the effect of the magnetic parameter M and volume fractions $\phi_{1},\phi_{2},\phi_{3}$ on Rex−1/2Nux and Rey−1/2Nuy when Pr=1 , Rex−1/2Nux , and Rey−1/2Nuy increase slowly. When nanoparticles are added to the base fluid, the effective viscosity of the hybrid nanofluid usually increases. Therefore, as the volume fractions of the nanoparticles increase, the skin friction curve increases. On the other hand, as the volume fractions of nanoparticles in the hybrid nanofluid increase, there are more interactions between the nanoparticles inside the hybrid nanofluid, resulting in a decrease in the distribution of the Nusselt number.

## 4. Conclusions

This article investigated the boundary-layer flows and heat transfer of three-dimensional viscous magnetohydrodynamic hybrid nanofluids using the homotopy analysis method. Similarity transformations were used to convert the interconnected governing PDEs of the problem into ODEs. The homotopy analysis method was utilized to obtain a series solution that is uniformly effective across the entire spatial region of 0 < *η* < ∞. The errors in the HAM calculation are smaller than 1 × 10^−9^ when compared to the results from the references. The volume fractions of the hybrid nanofluid and magnetic fields have significant impacts on the velocity and temperature profiles. This leads us to conclude that the appearance of magnetic fields can alter the properties of hybrid nanofluids, thereby altering the local reduced friction coefficient and Nusselt numbers. As the volume fractions of the nanoparticles increase, the effective viscosity of the hybrid nanofluid also typically increases, resulting in an increase in the local skin friction coefficient. The increased interactions between the nanoparticles in the hybrid nanofluid lead to a decrease in the distribution of the Nusselt number. In the future, the existing models can be extended and studied in different ways. Non-similarity transformation can be used to transform the governing equations into PDEs for the homotopy analysis method and compared with different numerical techniques, for example, the integrated simulation approach. The findings of this study may promote the development of effective heat transfer technologies and methods for using hybrid nanofluids, such as electronic cooling, energy systems, and aerospace engineering applications.

## Figures and Tables

**Table 1:** Thermophysical properties of H_2_O and nanoparticles [46,47,51].

Properties	H_2_O	$\mathrm{Cu}\;(\phi_{1})$	${\mathrm{Al}}_{2}{O_{3}}_{\;}(\phi_{2})$	${\mathrm{TiO}}_{2}\;(\phi_{3})$
$C_{p}\;\left(J/\mathrm{KgK}\right)$	4179	385	765	686.2
$\rho \;\left({\mathrm{Kg}/m}^{3}\right)$	997.1	8933	3970	4250
$\sigma \;\left(S/m\right)$	5.5 × 10^−6^	3.69 × 10^7^	5.96 × 10^7^	1 × 10^−18^
$k\;\left(W/\mathrm{mk}\right)$	0.613	400	40	8.9538

**Table 2:** A comparison of the 20th-order HAM-determined parameters λ=1 , ℏ=−0.7 when $\phi_{1}=\phi_{2}=\phi_{3}=0$ , M=0 , and Pr=1 , with Wang’s data [58].

c	$f^{\prime \prime }\left(0\right)$	$f^{\prime \prime }\left(0\right)$ [58]	$g^{\prime }\left(0\right)$	$g^{\prime \prime }\left(0\right)$ [58]	$f\left(\infty \right)$	$f\left(\infty \right)$ [58]	$g\left(\infty \right)$	$g\left(\infty \right)$ [58]
0	−1	−1	0	0	1	1	0	0
0.25	−1.04881	−1.04881	−0.19456	−0.19456	0.90715	0.90708	0.25799	0.25799
0.50	−1.09310	−1.09310	−0.46521	−0.46521	0.84239	0.84236	0.45168	0.45167
0.75	−1.13449	−1.13449	−0.79462	−0.79462	0.79230	0.79231	0.61214	0.61205
1	−1.17372	−1.17372	−1.17372	−1.17372	0.75150	0.75153	0.75150	0.75153

**Table 3:** HAM computations of various orders of the residual errors with $\phi_{1}=0.1$ , $\phi_{2}=0$ of the hybrid nanofluid Cu-Al_2_O_3_-H_2_O, when M=1 , Pr=1 , and c=0.5 ; determined parameters λ=0.6 , ℏ=−0.35 .

Mth-Order	$Err_{1}$	$Err_{2}$	$Err_{3}$	CPU Time (s)
1	0.15849	0.03162	0.00251	0.20313
5	0.01000	0.00250	0.00040	4.90625
10	0.00079	0.00015	0.00006	33.5156
15	3.98107 × 10^−5^	7.94328 × 10^−6^	6.30957 × 10^−6^	127.641
20	2.51189 × 10^−6^	7.94328 × 10^−7^	1.58489 × 10^−6^	325.484
25	2.51188 × 10^−7^	3.98107 × 10^−8^	2.51185 × 10^−7^	871.109
30	1.62181 × 10^−8^	3.98107 × 10^−9^	6.30957 × 10^−8^	4006.94

## Data Availability

All of the data produced during this study are contained within the article.

## Nomenclature

u,v,w	velocity components
T	temperature of the hybrid nanofluids
a,b	positive constants
$T_{\infty }$	ambient temperature
$T_{w}$	surface temperature
B	a uniform external magnetic field
k	thermal conductivity
$\rho C_{p}$	heat capacitance
$Cf_{x},Cf_{y}$	coefficients of skin friction
$Nu_{x},Nu_{y}$	Nusselt number
$\tau_{x},\tau_{y}$	shear stress
Pr	Prandtl number
Rex,Rey	local Reynolds numbers
ρ	fluid density
$\mu_{f}$	dynamic viscosity of the base fluid
$\phi ,\phi_{1},\phi_{2}$	volume fraction of the hybrid nanofluids
$\nu_{f}$	kinematic viscosity of the base fluid
$k_{hnf}$	thermal conductivity of the hybrid nanofluid
$\sigma_{hnf}$	electrical conductivity of the hybrid nanofluid
